# Factors associated with low birth weight at term: a population-based linkage study of the 100 million Brazilian cohort

**DOI:** 10.1186/s12884-020-03226-x

**Published:** 2020-09-14

**Authors:** Ila R. Falcão, Rita de Cássia Ribeiro-Silva, Marcia F. de Almeida, Rosemeire L. Fiaccone, Aline dos S. Rocha, Naiá Ortelan, Natanael J. Silva, Enny S. Paixao, Maria Yury Ichihara, Laura C. Rodrigues, Mauricio L. Barreto

**Affiliations:** 1 School of Nutrition, Federal University of Bahia, Salvador, Brazil; 2 Center for Data and Knowledge Integration for Health (CIDACS), Oswaldo Cruz Foundation, Salvador, Brazil; 3 School of Public Health, University of São Paulo, São Paulo, Brazil; 4 Department of Statistics, Institute of Mathematics, Federal University of Bahia, Salvador, Brazil; 5 Epidemiology and Population Health, London School of Hygiene and Tropical Medicine, London, UK; 6 Institute of Collective Health, Federal University of Bahia, Salvador, Brazil

**Keywords:** Low birth weight, Term birth, Poor populations, Cohort, Linkage

## Abstract

**Background:**

Factors associated with low birth weight at term (TLBW), a proxy for intrauterine growth restriction (IUGR), are not well-elucidated in socioeconomically vulnerable populations. This study aimed to identify the factors associated with TLBW in impoverished Brazilian women.

**Methods:**

Records in the 100 Million Brazilian Cohort database were linked to those in the National System of Information on Live Births (SINASC) to obtain obstetric, maternal, birth and socioeconomic data between 2001 and 2015. Multivariate logistic regression was performed to investigate associations between variables of exposure and TLBW.

**Results:**

Of 8,768,930 term live births analyzed, 3.7% presented TLBW. The highest odds of TLBW were associated with female newborns (OR: 1.49; 95% CI: 1.47–1.50), whose mothers were black (OR: 1.20; 95% CI: 1.18–1.22), had a low educational level (OR: 1.57; 95% CI: 1.53–1.62), were aged ≥35 years (OR: 1.44; 95% CI: 1.43–1.46), had a low number of prenatal care visits (OR: 2.48; 95% CI: 2.42–2.54) and were primiparous (OR: 1.62; 95% CI: 1.60–1.64). Lower odds of TLBW were found among infants whose mothers lived in the North, Northeast and Center-West regions of Brazil compared to those in the South.

**Conclusion:**

Multiple aspects were associated with TLBW, highlighting the need to comprehensively examine the mechanisms underlying these factors, especially in more vulnerable Brazilian populations, in order to contribute to the elaboration of health policies and promote better conditions of life for poor and extremely poor mothers and children.

## Background

Birth weight is strongly associated with infant morbidity and mortality, and is considered a predictor of immediate and future health status in newborns [1–4]. In 2015, 14.6% of all children (~ 20.5 million) were born with low birth weight (LBW); 91% of these births occurred in low- and middle-income countries [5]. In Brazil, the prevalence of LBW was estimated at 8.5% in 2017 [6], a rate very similar to that found in Latin America and the Caribbean (8.7%), which has not reduced significantly in the last 15 years [5].

Low birth weight, defined as less than 2500 g, may be a consequence of prematurity or associated with intrauterine growth restriction (IUGR), or a combination thereof [7]; The proportion of LBW related to restricted fetal growth and/or prematurity varies in accordance with the degree of economic development among countries [8]. In South Asia, among neonates with LBW, approximately 65% born at term were small for gestational age (a proxy for fetal growth restriction), while just over 50% presented LBW in Latin America and the Caribbean [8].

Evidence suggests that low birth weight at term (TLBW) (a proxy for IUGR) is associated with: i. maternal characteristics, such as age, and obstetrics history [9–15]; ii. newborn characteristics [10, 12]; iii. prenatal care [10, 11, 14]; iv. socioeconomic aspects [10, 12–14, 16–18]. Studies in low- and middle-income countries indicate that socioeconomic factors, including education, income, an urban/rural living environment, region of residence and domiciliary conditions, as well as access to prenatal care, are also important determinants of pregnancy and birth weight outcomes [4, 10–14, 16–23].

Although several studies have examined the determinants of TLBW, factors associated with TLBW remain unelucidated among socioeconomically vulnerable populations living in low- and middle- income countries, such as Brazil. To further investigate factors associated with TLBW, this study considered data between 2001 and 2015 from the 100 Million Brazilian Cohort linked to the National Live Birth System (SINASC). The 100 M Brazilian cohort contains information on low-income families with monthly per-capita income less than BRL200 (US$50), representing approximately 55% of the total Brazilian population [24]. Thus, the linkage of these two datasets enabled us to investigate the factors associated with TLBW in the Brazilian population living in poverty and extreme poverty, with the hope of contributing to the development of intervention strategies aimed at minimizing LBW.

## Methods

### Study design and population

This study employed data from the 100 Million Brazilian Cohort, a database constructed by the Center for Data and Knowledge Integration for Health (Centro de Integração de Dados e Conhecimentos para Saúde-CIDACS), affiliated with the Oswaldo Cruz Foundation (FIOCRUZ) [24]. This is a retrospective and dynamic cohort. The cohort database contains records of 114,001,661 low-income individuals (40,542,929 families) eligible for social assistance programs via the Unified Registry for Social Programs (CadÚnico), who were registered between between 2001 and 2015. Socioeconomic data from the 100 Million Brazilian Cohort were linked to data contained in the National System of Information on Live Births (SINASC), considering the period of Jan 1, 2001 to Dec 31, 2015.

Cohort data were linked to the live birth registry from SINASC according to similarity using the CIDACS Record Linkage algorithm [25]. This novel record linkage tool considers the following attributes in its matching process: mother’s name or newborn’s name, mother’s municipality of residence at time of registry/delivery, newborn date of birth and/or mother’s age. In the current linkage process, the number of linked records was 24,695,618 (55.51%) and the estimated accuracy was over 90%/year.

**Fig. 1:**
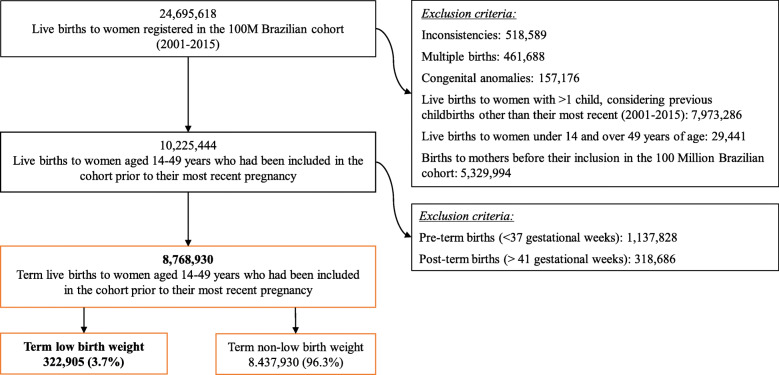
Flowchart description detailing obtainment of study population

The study population included live births of women aged 14–49 years who were registered in the 100 Million Brazilian Cohort between 2001 and 2015 (Fig. 1). We included only the most recent live birth reported for each woman, and excluded preterm (< 37 gestational weeks) and post-term births (42 gestational weeks or more). Multiple births and newborns with congenital anomalies were excluded in an effort to avoid bias, as these conditions are known to be strongly associated with low birth weight [13, 16, 26, 27].

### Variables

Descriptions for the variables of interest are detailed in Table 1. The dependent variable was low birth weight, defined as less than 2500 g, in term births (37–41 completed weeks) [28]. The following covariates were considered: i. socioeconomic characteristics (marital status, self-reported race/ethnicity, maternal schooling, household conditions, urban/rural living environment and geographic region of residence); ii. prenatal assistance (number of visits); iii. maternal- and newborn-related variables (maternal age at birth, newborn sex and birth order).

**Table 1:** Description of variables investigated in terms of associations with TLBW

Source	Classification	Variable	Description
SINASC	Dependent variable	Low Birth Weight	No (≥ 2.500g) Yes (< 2.500g)
100 Million Brazilian Cohort	Distal variables (Demographic and Socioeconomic characteristics)	Geographic region	South North Northeast Center-West Southeast
		Area of residence	Urban Rural
		Domiciliary conditions^a^	Appropriate Intermediate Inappropriate
		Self-declared maternal race/ethnicity	White/Yellow (Asian descent) Brown/Mixed-race (“*parda*”) Black Indigenous
SINASC	Distal variables (Demographic and Socioeconomic characteristics)	Marital status	Married, civil union Single, divorced, widowed
		Maternal schooling (years of formal education)	Illiterate 1 to 3 4 to 7 ≥ 8
SINASC	Intermediate variable (Prenatal assistance)	Number of prenatal visits	None 1 to 3 4 to 7 7 or more
	Proximal variables (Maternal and newborn characteristics)	Maternal age at time of childbirth (years)	14 to 20 20 to 35 35 to 49
		Newborn sex	Male Female
		Birth order (number of live childbirths including the current newborn)	1st child 2nd to 4th child 5th or later

^a^The domiciliary condition variable was created from the sum of the following six variables: building material (adequate: brick; inadequate: coated mud, wood, others), water supply (adequate: public network connection; inadequate: water well, spring, others), electricity (adequate: with meter for private or community use; inadequate: no meter), garbage disposal (adequate: city collection; inadequate: no collection, burned, buried, others), sewage (adequate: city public system; inadequate: others), and household density (adequate: ≤ 2 inhabitants per room; inadequate: > 2 inhabitants per room). Domiciliary conditions were considered as “adequate” when all variables were adequate; as “intermediate” when one or two variables were inadequate; and as “inadequate” when at least half (three or more) of the variables were considered inadequate

### Statistical analysis

**Fig. 2:**
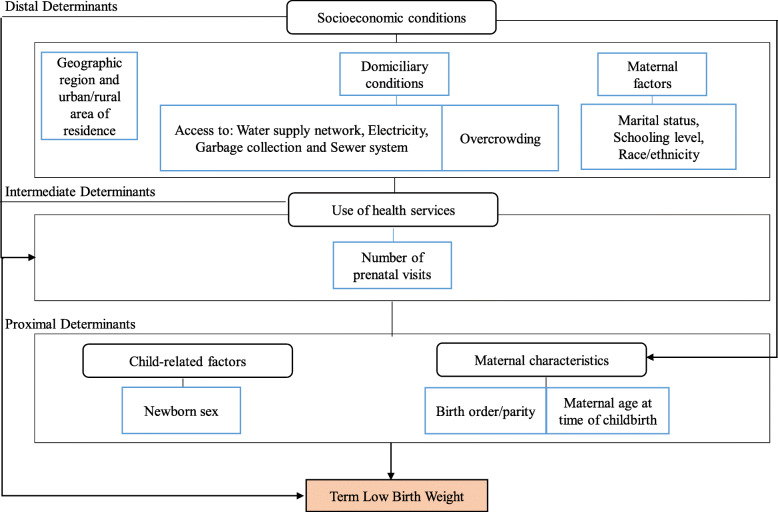
Conceptual hierarchy-based model used to analyze factors associated with term low birth weight

Maternal and live birth characteristics were summarized using frequency distributions. We also calculated the percentage of TLBW among all those born with LBW (6.7%), prior to excluding preterm and post-term births. Multivariate logistic regression was conducted to investigate the factors associated with TLBW. A conceptual hierarchy-based approach (Fig. 2) was employed to introduce, in subsequent adjusted models, the variables contained in the datasets, considering covariates deemed relevant and plausible in the literature [7, 9–18]. The TLBW variables were grouped into three blocks representing distal, intermediate and proximal determinants, i.e. socioeconomic characteristics, use of the health services (prenatal care, defined by number of visits) and maternal and newborn characteristics, respectively [7, 29–31].

The initial model was adjusted for the distal factors. In the second model, all variables contained in the previous model were maintained, with the inclusion of the number of prenatal visits (intermediate factor). The final model included, in addition to the variables contained in the two previous models, the mother’s age at the time of delivery, birth order/parity and sex of the newborn. Odds ratio (OR) values and respective confidence intervals (95% CI) were calculated for each variable maintained in the final model. Data analysis was performed using Stata version 15.1 (Stata Corporation, 153 College Station, USA).

### Advantages of large samples

Large sample sizes provide ample data to conduct analyses on subgroups of interest while maintaining sufficient power to gain insights into the direction and size of the effects. Due to changes in the SINASC registry with respect to gestational age, it was necessary to conduct additional analyses to verify the results of our multivariate regression analysis. From 2001 to 2010, gestational age was recorded as a categorical variable (gestational age in completed weeks: <22w; 22-27w; 28-31w; 32-36w; 37-41w; ≥42w). Beginning in 2011, gestational weeks at birth was recorded as a discrete variable (gestational age in number of completed weeks), estimated from the date of the mother’s last menstrual period, physical examination or image results obtained from SINASC records. Due to dynamic aspects of this cohort, such as the differences in the entry time of each woman in the study, as well as changes in 2011 in terms of how information on gestational age was collected/recorded by SINASC, additional analyses were carried out (shown in Suplemmentary material) for verification purposes. These additional multivariate logistic regression models were employed in accordance with the same variable selection method used in the main analysis, yet were stratified according to year of birth before and after 2011 and incorporated “time of exposure” quartiles prior to birth, considering the time difference (in years) between the mother’s inclusion in the cohort a newborn’s date of birth.

## Results

Among the 8,768,930 term live births investigated, 3.7% were found to be TLBW. Table 2 lists the characteristics of newborns with TLBW. The percentage of TLBW ranged slightly among different regions of Brazil, with a higher prevalence noted in the Southeast (4.0%) and South (3.8%) and similar prevalence among the Center-West, North and Northeast regions (3.5%). Regarding distal socioeconomic maternal characteristics, 53.9% of the mothers were unmarried (single, widowed or divorced), 59.4% had more than 8 years of schooling, 59.2% self-reported mixed-race (“parda”), 39.6% were born in the Northeast and 73.8% lived in urban areas.

**Table 2:** Characteristics of live births at term to women included in the 100 Million Brazilian cohort between 2001 and 2015 (*N* = 8,768,930)

Variables	Number of individuals with missing data (%)	Number of individuals (%)	LBW
			N (%)
Birth weight	8095 (0.1)		322,905 (3.7)
Geographic region	0 (0.0)		
South		1,026,468 (11.7)	39,022 (3.8)
North		936,916 (10.7)	32,677 (3.5)
Northeast		3,476,068 (39.6)	120,192 (3.5)
Southeast		2,734,958 (31.2)	110,157 (4.0)
Center-West		594,520 (6.8)	20,857 (3.5)
Area of residence	362,277 (4.1)		
Urban		6,207,608 (73.8)	230,689 (3.7)
Rural		2,199,045 (26.2)	78,624 (3.6)
Domiciliary conditions	653,514 (7.5)		
Appropriate		2,364,963 (29.1)	88,669 (3.8)
Intermediate		3,251,882 (40.1)	117,908 (3.6)
Inappropriate		2,498,571 (30.8)	92,577 (3.7)
Maternal race/ethnicity	734,010 (8.4)		
White/Yellow (Asian descent)		2,546,656 (31.7)	90,139 (3.5)
Brown/Mixed-race		4,758,109 (59.2)	173,876 (3.7)
Black		674,248 (8.4)	29,540 (4.4)
Indigenous		55,907 (0.7)	2168 (3.9)
Marital status	119,590 (1.4)		
Married, civil union		3,988,512 (46.1)	133,757 (3.4)
Single, divorced, widow		4,660,828 (53.9)	184,577 (4.0)
Maternal schooling (years)	166,492 (1.9)		
≥ 8		5,108,896 (59.4)	172,144 (3.4)
4 to 7		2,682,112 (31.2)	107,450 (4.0)
1 to 3		673,458 (7.8)	29,386 (4.4)
Illiterate		137,972 (1.6)	7437 (5.4)
Number of prenatal visits	66,725 (0.8)		
7+ visits		5,095,777 (58.6)	160,035 (3.1)
4 to 6 visits		2,834,653 (32.6)	115,592 (4.1)
1 to 3 visits		627,904 (7.2)	33,697 (5.4)
None		143,871 (1.7)	10,011 (7.0)
Maternal age at birth (years)	11,774 (0.1)		
20 to 35		6,072,560 (69.3)	201,913 (3.3)
14 to 20		1,755,503 (20.0)	76,544 (4.4)
35 to 49		940,218 (10.7)	44,426 (4.7)
Newborn sex	1363 (0.0)		
Male		4,475,726 (51.1)	134,850 (3.0)
Female		4,291,841 (49.0)	187,995 (4.4)
Birth order	531,511 (6.1)		
2nd to 4th child		4,847,199 (58.8)	151,366 (3.1)
5th or later		753,007 (9.1)	32,189 (4.3)
1st child		2,637,213 (32.0)	115,774 (4.4)

With regard to newborn/maternal characteristics, 69.3% of newborns’ mothers were aged 20–35 years, 32.0% had primiparous mothers, 58.8% had multiparous mothers (2–4 previous live births) and 49.0% were female.

**Fig. 3:**
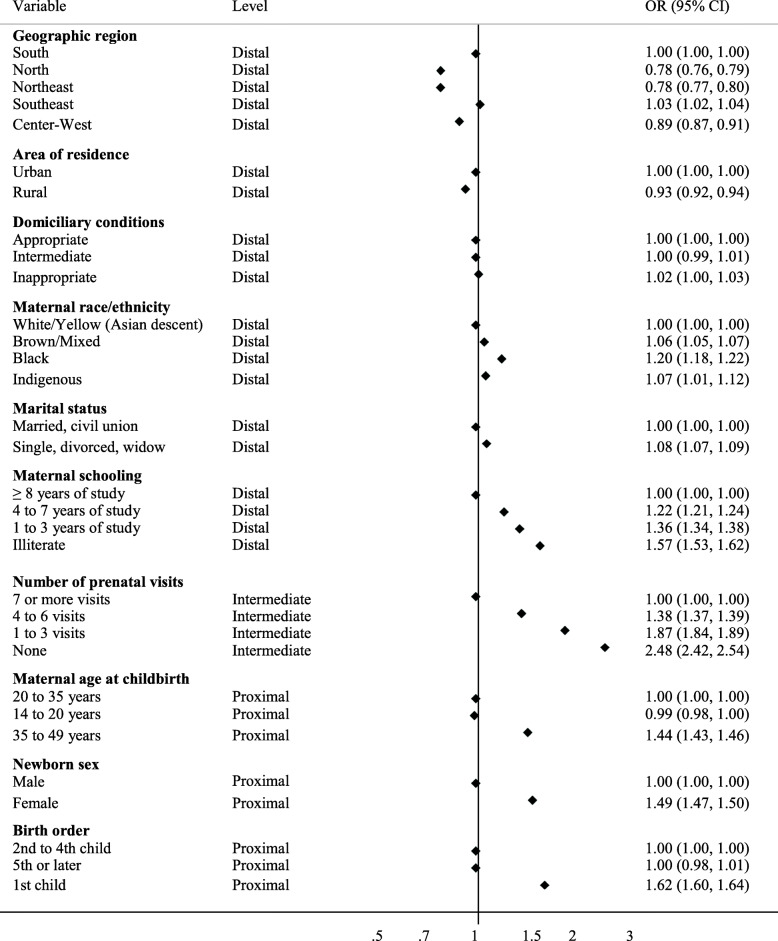
Adjusted model used to assess factors associated with term low birth weight

Figure 3 illustrates the results of our multivariate analysis. In the adjusted model, the odds of TLBW were higher among infants born to women who: self-declared skin color as black (OR: 1.20; 95% CI: 1.18–1.22), were unmarried (OR: 1.08; 95% CI: 1.07–1.09), had a low level of schooling (OR: 1.57; 95% CI: 1.53–1.62), had no prenatal visits (OR: 2.48; 95% CI: 2.42–2.54), were aged between 35 and 49 years (OR: 1.44; 95% CI: 1.43–1.46), gave birth for the first time (OR: 1.62; 95% CI: 1.60–1.64) and were female (OR: 1.49; 95% CI: 1.47–1.50). Dose-response associations were observed for the variables of schooling and number of prenatal visits. Lower odds of TLBW were observed among infants born to mothers living in the North (OR: 0.78; 95% CI: 0.76–0.79) and Northeast (OR: 0.78; 95% CI: 0.77–0.80) regions of Brazil. OR values approximating one were estimated for inadequate domiciliary conditions (OR: 1.02; 95% CI: 1.00–1.03) and a rural area of residence (OR: 0.93; 95% CI: 0.92–0.94). Our analysis of six models containing variables related to TLBW (supplementary material) confirmed the findings described above. The variables with stronger associations (self-reported race/ethnicity, level of schooling, age, number of prenatal visits, newborn sex and birth order) remained associated with TLBW in all supplementary models (Supplementary Tables 1 and 2).

## Discussion

The present study evaluated factors associated with TLBW in Brazilian populations living in poverty or extreme poverty. The occurrence of TLBW found herein was similar to that estimated in the overall Brazilian population born in 2017 (3.7%) [6]. Of all newborns with LBW, our results indicated that 55.2% were born at term, which is higher than in the overall population (38%) [6], illustrating the importance of TLBW with respect to the total burden posed by low birth weight in poorer populations. The occurrence of TLBW in our study approximated that reported in China (2.0%) [13] and rural China (4.8%) [32], yet was different from rates reported in Northern Ethiopia (10%) [9], at an Ethiopian hospital (12.0%) [33], at a Pakistani hospital (10.6%) [34] and in rural Central India (33.1%) [35]. Herein, lower rates of TLBW were observed among newborns whose mothers lived in the North and Northeast regions of Brazil. Moreover, our findings indicate that TLBW was associated with infants born to mothers with lower educational levels, who were black, unmarried, received an insufficient number of prenatal visits, were aged between 35 and 49 years, and whose newborns who were their first child and/or were female.

Previous studies in Brazil [36–39] have demonstrated that the country’s more developed regions, the South and Southeast, presented the highest percentages of children born with LBW. In contrast, a lower occurrence of LBW was found in the North and Northeast, which are considered economically less-developed regions; women in these areas also have lower levels of education, with notably less frequent or no prenatal consultations [36]. With respect to TLBW, these differences remain, yet are more subtle, as our results corroborate the higher frequency seen in the Southeast, being less common in the Northeast and Central-West regions [36].

It is worth noting that a demographic transition is well underway in Brazil, as evidenced by increasing fertility trends in women over 35 years of age, mainly in highly urbanized areas. This combined with the late onset of reproductive activity has led to an increase in the proportion of primiparous women in this age group [40]. In addition to delayed pregnancy, these results may also be explained by a lack of rigor in accurately recording live births at regional centers, as well as the precarious availability of health services and lack of early medical intervention [38].

Many studies have compared the occurrence of LBW among regions in Brazil, which hinders the ability to make comparisons regarding TLBW. A plausible explanation for discrepancies regarding rates of TLBW may be that, as some authors have observed, in locations with poor childbirth care resources and access to perinatal technology, newborns who die shortly after birth are commonly misclassified as “stillborn” or are not even registered [38, 39], which contributes to lower rates of LBW recorded in these areas, and may be a factor influencing the lower occurrence of LBW in northern and northeastern Brazil. Although not evaluated herein, another possibility might be higher maternal rates of smoking, which is a known cause of LBW [41, 42].

Our analysis indicated that TLBW is inversely associated with the level of maternal schooling, i.e. fewer years of formal study leads to a greater chance giving birth to a neonate with TLBW (Fig. 3). These findings corroborate those of many other studies investigating the factors associated with TLBW, highlighting the importance of socioeconomic conditions, especially with regard to mothers or their family’s educational level [10, 13, 14, 17, 18]. A mother’s level of schooling affects her use of health care services, as women with higher socioeconomic status often attend more prenatal visits, have better knowledge regarding nutrition [43] and generally understand and observe health professionals’ recommendations during pregnancy [44].

The findings of the present study also indicate that black women have a higher chance of giving birth to newborns with TLBW. How race translates into the social and economic environment and affects birth outcomes remains poorly understood [45]. While a previous study reported a higher occurrence of LBW in black women, this was only seen in individuals with low levels of education [3]. Another study found greater risk of LBW in Brazilian women of African descent (i.e. a grandparent, great grandparent, or great-great-grandparent born in Africa) [46]. We were unable to identify any studies reporting on racial disparities and TLBW in Brazil.

Marital status was found to be weakly associated with TLBW. It is possible that bias occurred in the recording of this variable, resulting in the overreporting of single women, since previous versions of the SINASC form used for registering live births did not include “stable union” as an option, rather specifying consensual union, which denotes a legal recognition of status [47]. However, this weak association did remain in our verification analysis (shown in Suplemmentary material) that compared study periods before and after 2011, the year in which changes were made to the live birth registry form, which further supports the observed association.

The estimated OR for TLBW was found to increase with reduced numbers of prenatal visits, indicating the importance of prenatal care. The provision of fewer prenatal health services, characterized by lower numbers of visits, has been associated with negative perinatal outcomes, such as TLBW [10, 11, 14]. The prevention of LBW is also conditional on compliance with nutritional guidance and lifestyle recommendations during pregnancy [43, 44], including the use of multivitamin supplements containing calcium, iron and folic acid, all micronutrients essential to proper fetal growth [29], in addition to the prevention of risk behaviors, e.g. use of tobacco, alcohol and other drugs [1, 45, 48–52].

Older women (between 35 and 49 years) are more likely to give birth to newborns with TLBW. Advanced maternal age, commonly defined as ≥35 years, is considered a risk factor for adverse health outcomes in children, including LBW and TLBW [9, 12–14, 18, 22, 23, 53–55], increased risk of comorbidities (hypothyroidism, type 2 diabetes, hypertension), obstetric complications (preeclampsia/eclampsia and emergency cesarean section) and the prior occurrence of obstetric complications in past pregnancies (history of spontaneous abortion and caesarean delivery) [56, 57]. Advanced maternal age has been particularly linked to LBW among primiparous women [57]. While extremes in maternal parity (primiparity and grand multiparity) are considered to present high risk of LBW and TLBW [14, 55], our results showed that only the first maternal pregnancy was associated with TLBW, which corroborates other findings in the literature [13, 18, 34]. As noted herein, some studies have reported increased risk for TLBW in female newborns [12, 18, 35], since male infants tend to have higher birth weights and face a lower risk of IUGR [7].

### Study strengths and limitations

The study has some important strengths. First, the large-scale dataset allowed us to comprehensively investigate known factors associated with TLBW. Second, the SINASC registry has high national coverage (over 90%), which provided a truly representative study population [58]. Moreover, the high reliability of the information contained in the SINASC database reinforces the suitability of using this system for epidemiological investigations [58]. With regard to limitations, the use of secondary data subjects the collected information to bias. This further implies that since data collection procedures were not performed as a function of the present study’s objectives, some important variables relevant to the determination of TLBW were not collected, such as maternal smoking habits, weight gain during pregnancy, the mother’s nutritional status and other maternal comorbidities. It is important to highlight that larger samples provide great opportunities for empirical research, but also may lead to equivocal interpretations due to the detection of statistical significance [59, 60].

This population-based study was focused on investigating live births to poor and extremely poor women. With due caution, some of the findings reported herein may be generalized to other populations with similar characteristics. Importantly, these limitations do not greatly detract from the promising potential enabled through research conducted on the “100 Million Brazilian Cohort”.

## Conclusion

This population-based study reveals important information regarding the frequencies and associated factors relative to TLBW in poor and extremely poor Brazilian women. Inequity in TLBW has persisted in economically vulnerable populations, especially in live births to mothers with lower educational levels who are black and receive insufficient prenatal care. The identification of factors associated with TLBW is essential to contribute to more inclusive health policies and promote improved welfare for poor and extremely poor mothers and children. Our study highlights the need to assess the underlying mechanisms behind these factors and expand on the analysis through the further exploration of the variables considered herein. We observed that a very high percentage of women receive an insufficient number of prenatal consultations, despite living in a country with a nationalized public health system. In sum, it will be important not only to implement social policies designed to protect pregnant women is economically vulnerable situations, such as through conditional cash transfer programs, but also to provide greater coverage of prenatal care as well as educational support regarding health and nutrition, in addition to paying special attention to the occurrence of comorbidities, especially among older women.

## Supplementary information

**Additional file 1: Table S1.** Adjusted models^†^ used to assess factors associated with term low birth weight by year of birth. **Table S2.** Adjusted models^†^ used to assess factors associated with term low birth weight by exposure time at birth categorized into quartiles intervals.

## Data Availability

All data supporting the findings presented herein were obtained from Centro de Integração de Dados e Conhecimentos para Saúde (CIDACS). Importantly, restrictions apply to the availability of these data, which were licensed for exclusive use in the current study, and are thus not publicly available. Upon reasonable request and with the express permission of CIDACS, the authors are willing to make every effort to grant data availability.

## Abbreviations

TLBW: Low birth weight at term
LBW: Low birth weight
IUGR: Intrauterine growth restriction
SINASC: National Live Birth System
OR: Odds ratio
95%CI: 95% confidence interval
