# Supplementary material for: Factors associated with low birth weight at term: a population-based linkage study of the 100 million Brazilian cohort
Source: BMC Pregnancy Childbirth. 2020 Sep 14;20:536. doi: 10.1186/s12884-020-03226-x (PMC7491100; doi:10.1186/s12884-020-03226-x)
Supplement: Supplementary file 1 — Additional file 1: Table S1. Adjusted models† used to assess factors associated with term low birth weight by year of birth. Table S2. Adjusted models† used to assess factors associated with term low birth weight by exposure time at birth categorized into quartiles intervals. [file 12884_2020_3226_MOESM1_ESM.docx]

**Appendices**

**Table 1.** Adjusted models^†^ used to assess factors associated with term low birth weight by year of birth.

| **Variable** | **Births prior to 2010 (N= 2,590,433)** | **Births in or after 2011 (N=4,610,320)** |
| --- | --- | --- |
|  | **OR (95% CI)** | **OR (95% CI)** |
| Geographic Region |  |  |
| South | Ref | Ref |
| North | 0.72 (0.70; 0.74) | 0.82 (0.80; 0.84) |
| Northeast | 0.72 (0.70; 0.73) | 0.83 (0.82; 0.85) |
| Southeast | 1.01 (0.99; 1.04) | 1.05 (1.03; 1.07) |
| Center-West | 0.82 (0.79; 0.85) | 0.93 (0.91; 0.95) |
| Area of residence |  |  |
| Urban | Ref | Ref |
| Rural | 0.93 (0.91; 0.94) | 0.96 (0.95; 0.97) |
| Maternal race/ethnicity |  |  |
| White/Yellow (Asian descent) | Ref | Ref |
| Mixed-race (*parda*) | 1.07 (1.06; 1.09) | 1.05 (1.03; 1.06) |
| Black | 1.22 (1.19; 1.25) | 1.18 (1.16; 1.20) |
| Indigenous | 1.05 (0.96; 1.14) | 1.06 (1.00; 1.12) |
| Marital status |  |  |
| Married, civil union | Ref | Ref |
| Single, divorced, widow | 1.13 (1.11; 1.14) | 1.06 (1.05; 1.07) |
| Maternal schooling (years) |  |  |
| ≥ 8 | Ref | Ref |
| 4 to 7 | 1.20 (1.18; 1.21) | 1.24 (1.23; 1.25) |
| 1 to 3 | 1.32 (1.29; 1.35) | 1.43 (1.40; 1.47) |
| Illiterate | 1.55 (1.50; 1.61) | 1.61 (1.54; 1.69) |
| Number of prenatal visits |  |  |
| 7 or more | Ref | Ref |
| 4 to 6 | 1.31 (1.29; 1.33) | 1.43 (1.42; 1.45) |
| 1 to 3 | 1.87 (1.82; 1.91) | 1.86 (1.83; 1.90) |
| None | 2.55 (2.46; 2.65) | 2.43 (2.36; 2.51) |
| Maternal age at birth (years) |  |  |
| 20 to 35 | Ref | Ref |
| 14 to 20 | 1.01 (0.99; 1.03) | 0.98 (0.97; 1.00) |
| 35 to 49 | 1.50 (1.48; 1.53) | 1.39 (1.37; 1.41) |
| Newborn's sex |  |  |
| Male | Ref | Ref |
| Female | 1.46 (1.44; 1.48) | 1.50 (1.48; 1.51) |
| Birth order |  |  |
| 2nd to 4th child | Ref | Ref |
| 5th or later | 0.99 (0.97; 1.01) | 1.01 (0.98; 1.03) |
| 1st child | 1.63 (1.60; 1.65) | 1.62 (1.60; 1.64) |

^†^Models with complete data adjusted for covariates and year of inclusion in the 100 Million Brazilian cohort

| **Table 2.**  Adjusted models^†^ used to assess factors associated with term low birth weight by exposure time at birth categorized into quartiles intervals. | | | | |
| --- | --- | --- | --- | --- |
| **Variable** | **Model 1 (<=1Q) (N= 1,829,886)** | **Model 2 (>1Q e <=2Q) (N= 1,834,668)** | **Model 3(>2Q e <=3Q) (N= 1,806,192)** | **Model 4 (>3Q) (N= 1,730,007)** |
|  | **OR (95% CI)** | **OR** | **OR** | **OR** |
| Geographic Region |  |  |  |  |
| South | Ref | Ref | Ref | Ref |
| North | 0.75 (0.72; 0.78) | 0.76 (0.73; 0.78) | 0.80 (0.78; 0.83) | 0.83 (0.80; 0.86) |
| Northeast | 0.75 (0.73; 0.77) | 0.76 (0.74; 0.78) | 0.81 (0.79; 0.84) | 0.82 (0.80; 0.84) |
| Southeast | 1.02 (1.00; 1.05) | 1.02 (0.99; 1.05) | 1.04 (1.02; 1.07) | 1.03 (1.01; 1.06) |
| Center-West | 0.84 (0.81; 0.87) | 0.87 (0.83; 0.90) | 0.91 (0.88; 0.95) | 0.94 (0.90; 0.97) |
| Area of residence | |  |  |  |
| Urban | Ref | Ref | Ref | Ref |
| Rural | 0.93 (0.91; 0.94) | 0.95 (0.93; 0.96) | 0.96 (0.94; 0.98) | 0.95 (0.94; 0.97) |
| Maternal race/ethnicity |  |  |  |  |
| White/Yellow (Asian descent) | Ref | Ref | Ref | Ref |
| Mixed-race (*parda*) | 1.06 (1.04; 1.08) | 1.08 (1.05; 1.10) | 1.05 (1.03; 1.07) | 1.04 (1.02; 1.06) |
| Black | 1.19 (1.16; 1.22) | 1.23 (1.20; 1.27) | 1.20 (1.17; 1.24) | 1.17 (1.13; 1.21) |
| Indigenous | 1.11 (1.01; 1.22) | 1.14 (1.05; 1.25) | 1.08 (0.98; 1.18) | 0.93 (0.84; 1.03) |
| Marital status |  |  |  |  |
| Married, civil union | Ref | Ref | Ref | Ref |
| Single, divorced, widow | 1.10 (1.08; 1.11) | 1.10 (1.08; 1.11) | 1.06 (1.04; 1.08) | 1.07 (1.05; 1.09) |
| Maternal schooling (years) |  |  |  |  |
| ≥ 8 | Ref | Ref | Ref | Ref |
| 4 to 7 | 1.21 (1.19; 1.23) | 1.21 (1.19; 1.23) | 1.23 (1.21; 1.25) | 1.26 (1.23; 1.28) |
| 1 to 3 | 1.32 (1.28; 1.36) | 1.35 (1.31; 1.38) | 1.40 (1.36; 1.45) | 1.47 (1.42; 1.52) |
| Illiterate | 1.51 (1.44; 1.58) | 1.57 (1.49; 1.66) | 1.65 (1.55; 1.76) | 1.71 (1.59; 1.83) |
| Number of prenatal visits |  |  |  |  |
| 7 or more | Ref | Ref | Ref | Ref |
| 4 to 6 | 1.33 (1.31; 1.35) | 1.37 (1.34; 1.39) | 1.40 (1.37; 1.42) | 1.45 (1.43; 1.48) |
| 1 to 3 | 1.82 (1.77; 1.87) | 1.85 (1.80; 1.90) | 1.90 (1.85; 1.96) | 1.90 (1.85; 1.96) |
| None | 2.39 (2.29; 2.51) | 2.59 (2.47; 2.72) | 2.50 (2.38; 2.62) | 2.48 (2.36; 2.60) |
| Maternal age at childbirth (years) |  |  |  |  |
| 20 to 35 | Ref | Ref | Ref | Ref |
| 14 to 20 | 1.00 (0.98; 1.02) | 1.00 (0.97; 1.02) | 0.99 (0.97; 1.02) | 0.97 (0.95; 1.00) |
| 35 to 49 | 1.44 (1.41; 1.48) | 1.41 (1.38; 1.44) | 1.46 (1.42; 1.50) | 1.42 (1.38; 1.46) |
| Newborn sex |  |  |  |  |
| Male | Ref | Ref | Ref | Ref |
| Female | 1.48 (1.46; 1.5) | 1.47 (1.44; 1.49) | 1.50 (1.48; 1.52) | 1.49 (1.47; 1.52) |
| Birth order |  |  |  |  |
| 2nd to 4th child | Ref | Ref | Ref | Ref |
| 5th or later | 1.02 (0.99; 1.05) | 1.00 (0.97; 1.03) | 0.99 (0.96; 1.02) | 0.96 (0.93; 0.99) |
| 1st child | 1.68 (1.64; 1.71) | 1.62 (1.59; 1.66) | 1.63 (1.60; 1.66) | 1.58 (1.55; 1.61) |

^†^Models with complete data adjusted for covariates and year of inclusion in the 100 Million Brazilian cohort
